# Gastrointestinal involvement in Parkinson’s disease: pathophysiology, diagnosis, and management

**DOI:** 10.1038/s41531-022-00295-x

**Published:** 2022-03-24

**Authors:** T. Warnecke, K-H. Schäfer, I. Claus, K. Del Tredici, W. H. Jost

**Affiliations:** 1 Department of Neurology with Institute of Translational Neurology, University Hospital of Münster, 48149 Münster, Germany; 2 Research and Transfer Working Group Enteric Nervous System (AGENS), University of Applied Sciences Kaiserslautern, Campus Zweibrücken, 66482 Zweibrücken, Germany; 3 Clinical Neuroanatomy, Department of Neurology, Center for Biomedical Research, University of Ulm, 89081 Ulm, Germany; 4 Parkinson-Klinik Ortenau, 77709 Wolfach, Germany

**Keywords:** Parkinson's disease, Neurodegeneration

## Abstract

Growing evidence suggests an increasing significance for the extent of gastrointestinal tract (GIT) dysfunction in Parkinson’s disease (PD). Most patients suffer from GIT symptoms, including dysphagia, sialorrhea, bloating, nausea, vomiting, gastroparesis, and constipation during the disease course. The underlying pathomechanisms of this α-synucleinopathy play an important role in disease development and progression, i.e., early accumulation of Lewy pathology in the enteric and central nervous systems is implicated in pharyngeal discoordination, esophageal and gastric motility/peristalsis impairment, chronic pain, altered intestinal permeability and autonomic dysfunction of the colon, with subsequent constipation. Severe complications, including malnutrition, dehydration, insufficient drug effects, aspiration pneumonia, intestinal obstruction, and megacolon, frequently result in hospitalization. Sophisticated diagnostic tools are now available that permit more detailed examination of specific GIT impairment patterns. Furthermore, novel treatment approaches have been evaluated, although high-level evidence trials are often missing. Finally, the burgeoning literature devoted to the GIT microbiome reveals its importance for neurologists. We review current knowledge about GIT pathoanatomy, pathophysiology, diagnosis, and treatment in PD and provide recommendations for management in daily practice.

## Introduction

Thirty years ago, gastrointestinal tract (GIT) symptoms in Parkinson’s disease (PD) played a subordinate role in clinical practice of most neurologists and movement disorders specialists, despite the existence of numerous very early clinical reports and neuropathological findings that clearly provided evidence of relevant GIT involvement^1–3^. Although GIT dysfunction can precede somatomotor symptoms by up to 20 years^4–6^ and impact negatively on quality of life^7,8^, clinicians mainly focused on the ‘classical’ motor symptomatology at that time. It was not until the late 1980s and early 1990s that the number of clinical studies and neuropathology publications about the complex interaction of PD and the GIT begin to increase, thereby, also stimulating the interest of clinical neurologists for this topic^9–15^. After 2000, the quest for explanations regarding the potential role of the GIT and the peripheral nervous system in the pathogenesis of PD gained momentum owing to the work by the Braak group, among others^16–19^. Thus, one finds in PubMed for the period 1960–1969 eleven (keywords ‘Parkinson’ and ‘dysphagia’) and four (keywords ‘Parkinson’ and ‘constipation’) publications, from 1970 to 1979 twenty (‘Parkinson’ and ‘dysphagia’) and seven (‘Parkinson’ and ‘constipation’) publications, as opposed to more than ten entries per year beginning in 1992 and 2003, and, alone for the year 2020, 109 articles (‘Parkinson’ and ‘dysphagia’) and 93 (‘Parkinson’ and ‘constipation’) (<parkinson dysphagia - Search Results - PubMed (nih.gov) and< parkinson constipation - Search Results - PubMed (nih.gov)).

Today, GIT research is a promising and still growing field of inquiry and continues to provide neurologists and movement disorders specialists with novel and valuable data that can help to better understand the complexity of PD. At this point, we still have a multitude of jigsaw puzzle pieces that must be carefully pieced together. Periodic review articles are intended to serve as the basis for future work^5,6,20,21^. Here, we provide an overview of the latest knowledge, hypotheses, and debates about the pathology, pathophysiology, diagnostic methods for oropharyngeal and esophageal affection as well as impairment of the lower GIT, including a summary of current treatment strategies from an interdisciplinary standpoint. These might be helpful for neurologists, speech- and language therapists, and other clinicians in their daily work with PD patients and PD-associated GIT dysfunction.

## Anatomy: gastrointestinal tract and associated brain areas: central control of gastrointestinal motility

The entire GIT is one of the major gateways for extrinsic influences upon the human body. It is autonomously innervated by the largest part of the peripheral nervous system, the so-called enteric nervous system (ENS), which contains several hundreds of neurons^22^ and even more glial cells. Both neurons and glial cell populations have a variety similar to that found in the brain. Neurons consist of motoneurons, secretomotor-, or interneurons that express acetylecholine, nitric oxide synthase, catecholamines, GABA, or a broad range of neuropeptides^23–26^. Glial cells can be found in at least four different morphologies and chemical codings, expressing S100B, the reactive gliosis marker GFAP, PDGFRα, or proteolipid-protein-1^27,28^. Both neurons and glial cells form complex networks that populate in ganglionic and aganglionic plexus the complete gut wall from esophagus to anus and from serosa to mucosal layer.

The ENS varies significantly along the gut axis, analogue to the distinct functional differences between the individual gut segments. Different neuronal subtypes and glial cells form neuronal circuits that allow the autonomous regulation of gastrointestinal motility^29^. Although the gut works independently, there is a varying influence of the central nervous system (CNS) via several additional extrinsic inputs, of which the vagus nerve is the largest. The vagus nerve is part of the so-called brain-gut-axis that connects the CNS with the GIT. The brain-gut-axis consists of two main routes between the two organs^30^. One is based on humoral factors, such as cytokines, hormones, or even bacterial metabolites from the gut microbiome, whereas the other is a hard-wired connection: the vagus nerve. The vagus nerve contains up to 50,000 fibers that run in both directions, the afferent ones being the majority with ~90% of all fibers^31^. While the afferent fibers deliver information from the gut, the efferent fibers provide parasympathetic motor stimuli that originate in two brainstem nuclei, first the dorsal motor nucleus of the vagus and, second, the ambiguus nucleus, which both contribute to gastrointestinal motility. Additionally to this direct input from the brainstem, there are several routes of influence represented, i.e., by sympathetic fibers from prevertebral ganglia that connect the gut with thoracic segments of the spinal cord^29^.

The CNS influence upon gastrointestinal motility is dependent on the location. While there is a considerable impact on both esophagus and stomach^32^, the gut becomes more independent in the small and large intestine, where autonomous reflex circuits control smooth muscle activity, local blood flow, or secretion and absorption along the mucosal barrier. Interestingly, recent studies provide evidence that there are neural connections between the vagal nuclei and various areas of the cortex that influence stomach motility^33^ and, thus, might be affected in PD, as demonstrated in a rat model^34^. Especially the latter might explain the top-down gastrointestinal symptoms in PD. Based on the dual-hit hypothesis^35^, there will also be a bottom-up process, possibly initiated by local inflammation and a compromised mucosal barrier, that allows gut content, including lipopolysaccharides, short chain fatty acids (SCFA) or other bacterial metabolites to enter the gut wall. In PD patients, the mucosal barrier is compromised and corresponding markers, such as calprotectin, can be found in the feces^36^. There is a vast amount of evidence that the microbiome in PD patients is disturbed^37^, combined with an alteration of SCFAs^38^. Recent studies demonstrate the existence of neuronal circuits that monitor the microbiome or its metabolites report to the CNS or lead to modification of the innervation^39^. These findings open up perspectives for using the gut, its intrinsic nervous system, the mucosal barrier, or the microbiome as therapeutic targets.

## Pathology: alpha-synucleinopathy in the GIT of incidental Lewy body disease and Parkinson’s disease

Lewy pathology (LP, Lewy bodies, Lewy neurites) in prodromal PD (at autopsy, incidental Lewy body disease, ILBD^40–42^) and in sporadic PD occurs throughout the human GIT^3,9,43–48^. Interpretation of ENS histological slides from intestinal biopsies requires caution because immunocytochemical protocols vary considerably, and α-synuclein immunoreactivity must be distinguished from α-synuclein aggregates (LP) and α-synuclein aggregating species^49^. As staged cases show, LP exists in the olfactory bulb, spinal cord, peripheral autonomic ganglia, submandibular gland, cardiac nerves, and ENS before it appears in the substantia nigra, pars compacta, and before neuronal loss occurs there^17,50–54^. The aggregated α-synuclein lesions are not transient.

One of the largest studies examined a wide range of organs from 92 autopsied individuals, including 17 PD, 7 ILBD, and 23 controls^46^. In pure PD, LP was found in the GIT of 64.7% (11/17), peripheral vagal nerve (pN. *X*) of 73.3%, and sympathetic trunk of 80% of cases. In ILBD, 14.2% (1/7) showed LP in the GIT and 28.57% in the pN. *X*. 50% of the ILBD group also displayed LP in the sympathetic trunk. A decreasing ENS rostral-caudal immunostaining gradient was seen, corroborating an earlier report^9^: The upper GIT, i.e., distal esophagus followed by the stomach, tended to display the highest pathological burdens^46^; both sites are directly controlled by parasympathetic preganglionic fibers of the vagus nerve^31,55,56^ (see section “Anatomy: gastrointestinal tract and associated brain areas: central control of gastrointestinal motility” above). Although there were no ‘ENS-only’ (i.e., ‘ENS-first’) cases in the cohort, the authors surmised that “the findings of the present study are not incompatible with a GI entry for PD, ILBD and DLB”^46^. In a subsequent investigation of pN. *X* tissue, they concluded that “the results [i.e., LP in 10/18 ILBD and 42/44 PD subjects; no LP in 49 controls] support initiation of Lewy-type alpha-synucleinopathy in the brain, with early, in some cases preclinical, subsequent progression to the peripheral nervous system”^57^. However, a lack of α-synuclein immunopositivity in the true control (as opposed to ILBD) group is not surprising [see also^58^] and, in any event, in 55.56% (ILBD) and 95.45% (manifest PD) of cases the pN. *X* was involved.

The 2010 study may have a selection bias because, to assess the relative LP frequency in the periphery, the ultimate choice of regions for further study was reduced to those with “a greater likelihood to have positive staining”^46^ rather than shared innervation or neuronal circuitries. In addition, for the majority of their cases, the authors examined only a single slide for each ENS subdivision^46^. Relevant ENS-related autonomic structures, e.g., the lumbar prevertebral celiac ganglion (sympathetic innervation of the esophagus, stomach, duodenum, pancreas, liver) and spinal cord SPS^44^, but also the appendix vermiformis and superior mesenteric ganglion^47,48,59^, were not included.

Results from investigations performed to date on large cohorts show, despite divergent findings from smaller studies^60,61^, that 0% of ILBD cases displayed LP in the ENS in the absence of brain lesions^44–46^. Of the prodromal PD cases analyzed by Stockholm et al., 44% (17/39) of subsequent PD patients displayed no ENS LP^47^, and not even all manifest PD cases from autopsy-based studies had ENS involvement^44,46,53,59^ see also ref. ^62^. The heterogeneity of findings obtained from such studies is complicated not only by differences in immunocytochemical staining techniques and study design (cohort sampling size and stratification, retrospective vs. longitudinal), but also by dissection protocols. Borghammer & Van Den Berge point out that the human GIT “measures ~8–10 m at post-mortem and has a geometric surface area of at least 7000 cm^2^. Thus, many hundreds of microscopy slides are required to rule out… gut pathology with any degree of confidence”^63^. Similarly, pN. *X* specimens necessarily come from a very limited portion of the entire nerve^44,52,57,58,64^, and multiple sections from both vagal trunks would be required to ascertain whether LP is present or absent in a given individual.

The argument that autopsy-based studies report only rare cases of LP in the ENS in the absence of brain LP does not eliminate the possibility of an ENS and/or peripheral nervous system origin for PD (ILBD), or of LP spread from the ENS to the brain^16,17,35,62^. That a potential anatomical pathway from intrinsic ENS neurons to the pN. *X* exists, receives support from the fact that the myenteric plexus, epithelial enteroendocrine cells, and preganglionic portions of the N. *X* express normal α-synuclein^65,66^. Converging lines of evidence also support the idea of propagation via cell-to-cell transsynaptic transmission of misfolded α-synuclein into recipient cells; there, misfolded α-synuclein can recruit native α-synuclein and become a template for development of pathological aggregates^67–69^. Formalin-fixed tissue from the stomach is capable of limited to robust seeding in ILBD (5/8 cases), PD (10/12 cases), and controls (2/9)^70^.

**Fig. 1:**
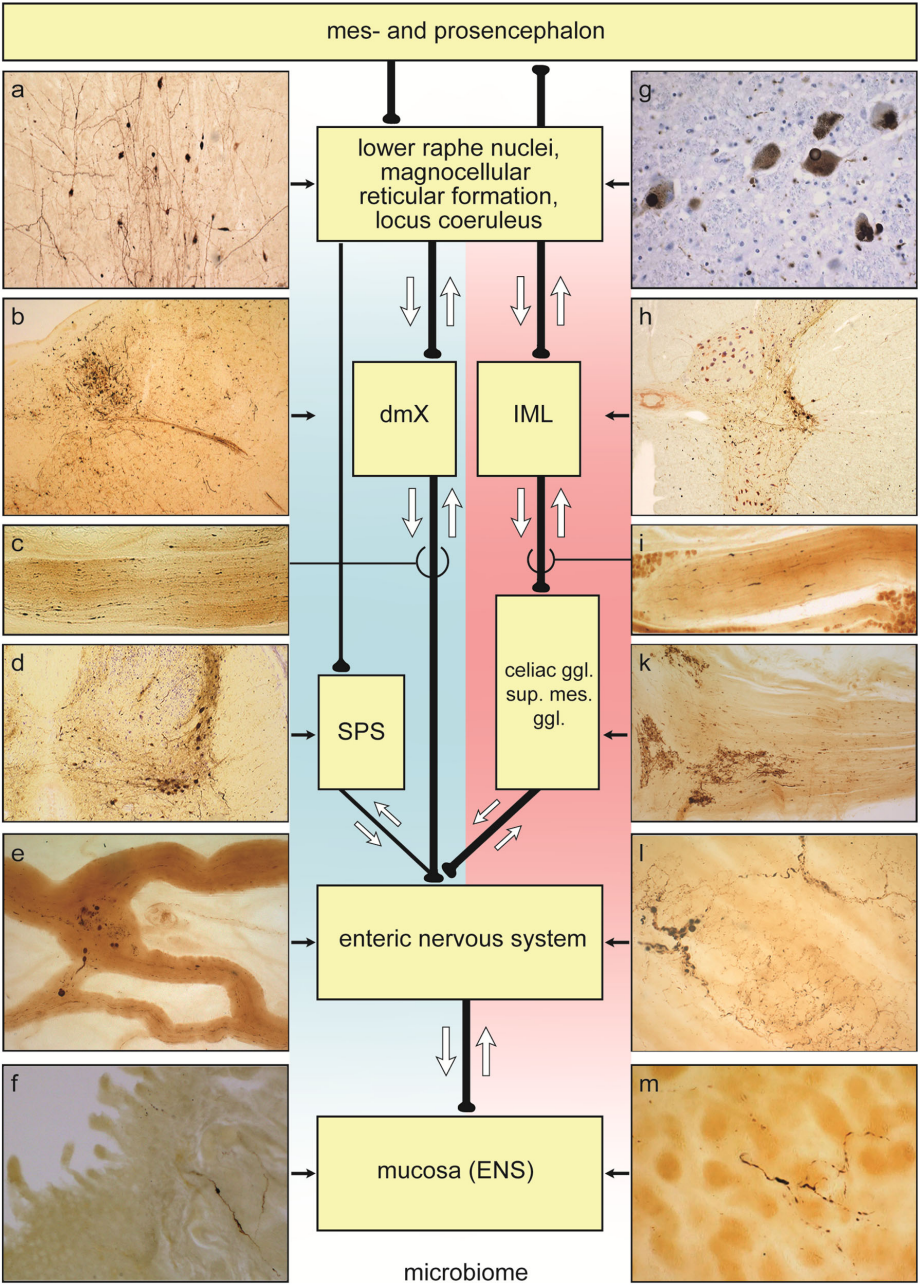
Diagram showing possible bidirectional (*white arrows*) parasympathetic (*blue background*) and sympathetic (*pink background*) pathways along which pathological α-synuclein propagation in ILBD and PD could occur between the periphery, including the ENS, and the CNS. *Retrograde*: *parasympathetic* (distal esophagus/stomach → pN. *X* → dorsal motor nucleus of the vagus nerve, dmX); *parasympathetic* (appendix vermiformis → RIM → pN. *X* → dmX); *parasympathetic* (descending colon and further distal → ganglion pelvicum → SPS preganglionic neurons → lower brainstem level-setting nuclei); *sympathetic* (distal esophagus/stomach → prevertebral celiac ganglion postganglionic neurons → IML preganglionic neurons → lower brainstem level-setting nuclei). Alternatively, *anterograde: parasympathetic* (dmX → pN. *X* → distal esophagus/stomach); *parasympathetic* (lower brainsteim level-setting nuclei → SPS preganglionic neurons → prevertebral postganglionic ganglion pelvicum → descending colon and portions further distal); *sympathetic* (appendix vermiformis → RIM → prevertebral SMG postganglionic neurons → Nn. splachnici → IML preganglionic neurons → lower brainstem level-setting nuclei).Abbreviations: pN. *X* peripheral vagus nerve, dmX dorsal motor nucleus of the vagus nerve, IML intermediate mediolateral nucleus, SPS sacral parasympathetic nucleus, RIM root of the small intestine mesentery, sup. mes. ggl. superior mesenteric ganglion. The level-setting nuclei consist of the lower raphe nuclei, magnocellular nucleus of the reticular formation, and locus coeruleus^81^. The RIM contains parasympathetic and sympathetic fibers innervating the upper GIT extending from the proximal jejunum to the distal ileum, thereby making it another potentially useful structure for neuropathological diagnosis of the existence of LP in the small intestine^61,232^. Illustrations showing LP in **a**–**m** are not to scale: **a** great raphe nucleus. **b** dmx and intramedullary N. *X*. **c** pN. *X* at level of the carotid bifurcation. **d** SPS. **e** Gastric cardia, Auerbach plexus, tangential section. **f** Jejunum, Meissner (submucous) plexus, transversal section. **g** Locus coeruleus. **h** IML. **i** Splanchnic nerve at the level of the celiac ganglion. **k** Celiac ganglion. **l** Distal esophagus, Auerbach plexus, tangential section. **m** Gastric cardia, Meissner (submucous) plexus, tangential section. LP within the lamina propria reach the mucosa near gastric glands. Syn-1 immunohistochemistry (BD Biosciences, Eysins, Switzerland) in 100–150 µm sections.

Overexpressed human α-synuclein and human α-synuclein lysates in animal models can mediate cellular dysfunction^71,72^ as well as pathological spreading (seeding) along the pN. *X* bidirectionally^73–75^, and anatomical connectivities^73,76–82^ make both directionalities conceivable in human α-synucleinopathy (Fig. 1). The latest findings in the appendix vermiformis of 46/48 ILBD cases^47,48^ indicate that additional ‘conduits’ for such pathological α-synuclein transport could exist (Fig. 1).

An awareness is gradually emerging that the presence of LP in the GIT and its patterns of progression may well differ between various PD subpopulations and in ILBD^48,58,63,83,84^. Borghammer & Van Den Berge postulated the existence of a ‘PNS-first Lewy body disorder phenotype’, wherein early pathology in the peripheral autonomic nervous system might spread along retrograde connectivities to nuclei of the lower brainstem that contribute to rapid-eye-sleep (REM) regulation^63^ (Fig. 1). This intriguing hypothesis, drawing on insights gleaned from idiopathic REM sleep behavioral disorder (RBD) research^85–92^, could also be tested neuropathologically, provided tissue from RBD patients were still available, in autopsy cohorts staged according to the 2003 PD staging protocol and including early ILBD^93–95^.

## Neurology: upper GIT

### Oropharyngeal phase

#### Prevalence of dysphagia

Oropharyngeal dysphagia is a common and often disabling clinical manifestation in PD. Recent meta-analysis estimates the prevalence of oropharyngeal dysphagia up to 82% during the course of the disease^96^. However, only 20–40% of patients are aware of their swallowing dysfunction, and only less than 10% report their complaint spontaneously^97,98^ which might result from early pharyngeal hyposensibility^21,99^. Recent research has led to the conclusion that oropharyngeal dysphagia is not only a late-stage PD symptom but can occur during any stage of the disease, including the preclinical or prodromal stages^96,100,101^. Therefore, a comprehensive examination of oropharyngeal swallowing function should be performed regularly, even in early disease stages, when defined clinical predictors (see below) are present^102^.

#### Pathophysiology

In contrast to all other parts of the GIT, the oropharynx is not only innervated by the involuntary ENS but is also controlled from voluntarily triggered mechanisms of skeletal muscle movements. Thus, some additional pathophysiological mechanisms play a role that are also relevant for other somatomotor symptoms of PD, such as bradykinesia or tremor^21^:

1. Accumulation of Lewy pathology (see section “Pathology: alpha-synucleinopathy in the GIT of incidental Lewy body disease and Parkinson’s disease” above) takes place not only in the substantia nigra but also in various non-dopaminergic swallowing-relevant brainstem and cortical areas.
2. Putamen and globus pallidus are activated bilaterally during normal swallowing. Therefore, lack of dopamine in the striatum of PD patients may impair this part of the supramedullary swallowing network.
3. Peripheral mechanisms might be also involved as indicated by α-synuclein deposits in the peripheral sensory and motor nerves of the larynx as well as disease-induced neuromuscular alterations of pharyngeal muscles^103–105^. Substance P plays an important role in these peripheral oropharyngeal mechanisms.

#### Substance P (SP)

SP is an ubiquitous neuropeptide in the nervous system, with immunoactive fibers having been detected in the laryngeal nerves, epithelium, and basal membrane of pharyngeal mucosa, especially on the surface of the epiglottis. SP mediates the response to local stimuli in the pharyngeal mucosa and thereby enhances the swallow and cough reflexes^106^. A reduction of substance P, found in PD patients’ sputum, is postulated to lead to a disturbance of protective reflexes and, ultimately, silent aspiration^107^. Reduced saliva concentrations of substance P may also be a predictor for the presence of early pharyngeal swallowing dysfunction^106^. Table 1 provides an overview of PD-related oropharyngeal dysphagia clinical manifestations and postulated pathomechanisms^108^.

**Table 1:** Overview of PD-related oropharyngeal dysphagia clinical manifestations and postulated pathomechanisms.

Clinical manifestation	Pathomechanisms
Prolonged oral transit time:	Dopaminergic + non-dopaminergic (especially Lewy pathology in swallowing cortex?)
Premature spillage:	Dopaminergic + non-dopaminergic (Lewy pathology in swallowing cortex?)
Delayed swallow reflex:	Dopaminergic + decreased Substance P concentration
Prolonged pharyngeal transit time:	Dopaminergic + non-dopaminergic (Lewy pathology in brainstem?)
Penetration:	Dopaminergic + non-dopaminergic
Aspiration:	Dopaminergic + non-dopaminergic
Residue in valleculae:	Primarily dopaminergic
Residue in piriform sinus:	Dopaminergic + non-dopaminergic
Dysfunction of upper esophageal sphincter:	Primarily non-dopaminergic (Lewy pathology in swallowing centers of medulla oblongata?)
Insufficient cough reflex:	Decreased Substance P concentration

Source^108^.

#### Main pathological findings

The main phenotype characteristic of PD-related dysphagia is insufficient pharyngeal bolus clearing, with residues predominant located in the valleculae, in addition to pharyngolaryngeal movement disorders, above all pharyngeal bradykinesia^109^. Silent aspiration is also a frequent clinical manifestation, even in early stages, but the risk increases with disease duration^100^. In an analogy to freezing of gait with similar pathophysiologic mechanisms, a recent study described the presence of oropharyngeal freezing resulting in a temporally missing or delayed swallowing reflex^110^.

**Fig. 2:**
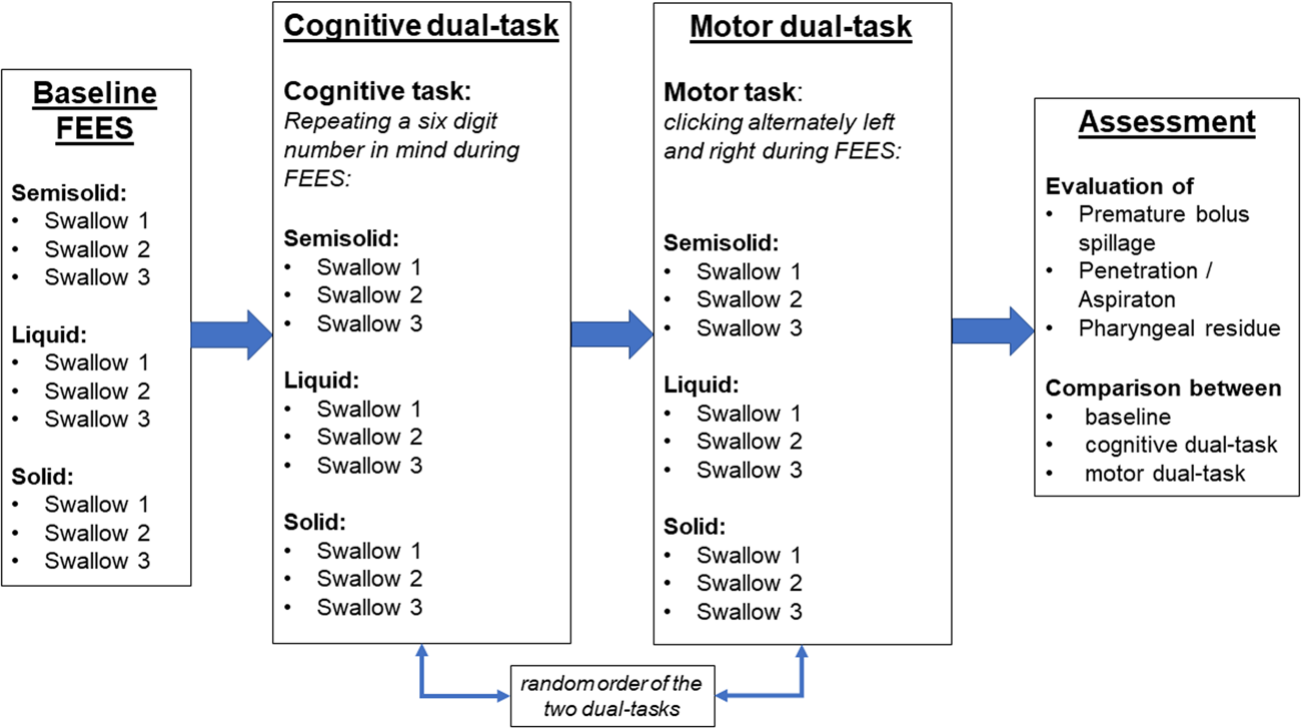
Dual Task examination algorithm via Flexible Endoscopic Evaluation of Swallowing (FEES) (adapted from ref. ^112^). FEES examination protocol including cognitive and motor dual-task for evaluation of swallowing function in PD patients.

**Fig. 3:**
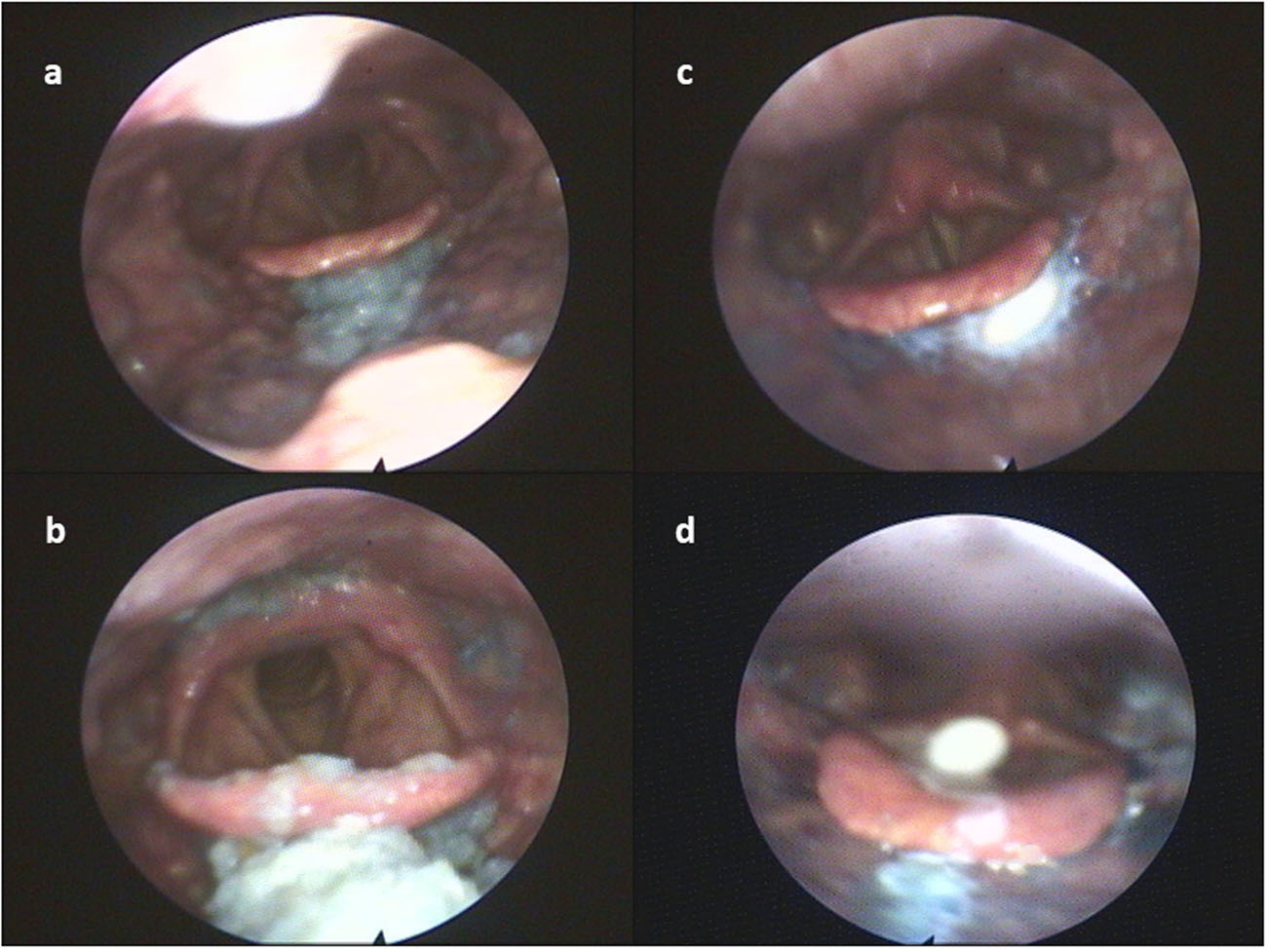
Examples for pharyngeal residue via FEES. **a** Mild residue for solid food located in the valleculae. **b** Moderate to severe residue for solid food located in the valleculae and piriform sinus with penetration into the laryngeal vestibule. **c** Tablet residue located in the valleculae. **d** Tablet penetration.

Dual task situations (i.e., cognitive or somatomotor tasks) are challenging to swallowing functional reserve capacities and therefore should be integrated into standard instrumental swallowing evaluations^111,112^ (Fig. 2). Furthermore, retention of medications in the hypopharynx for long periods of time may account for erratic absorption of levodopa with an insufficient or unpredictable clinical response to oral medication^21^ (Fig. 3). An association of delayed on-phenomena with pharyngeal residues could have been shown as well^113^. In a recent study, substantially impaired ability to swallow tablets or capsules was found in 28% (*n* = 33/118) of patients at all stages of the disease^114^. Capsules were the easiest to swallow, whereas oval tablets were the most difficult^114^.

#### Clinical predictors

The following clinical conditions have been linked to oropharyngeal dysphagia in PD and can be considered predictors^21,115,116^:

- Hoehn and Yahr stage ≥III
- Relevant weight loss
- Body Mass Index (BMI) ≤20 kg/m^2^
- Severe drooling or sialorrhea
- Dementia

Another major problem for PD patients is drooling^117,118^. Sialorrhea in PD patients usually does not result from an increased production of saliva but from a reduced spontaneous swallowing rate (48/h vs. 71/h) and/or from oropharyngeal dysphagia with a reduced ability to swallow saliva^52^. The extent of sialorrhea correlates with the severity of PD-related dysphagia^119^.

### Diagnostic management

#### Screening-tools

Questionnaires and a specific water swallow test may be used as screening tools:

1. The Swallowing Disturbance Questionnaire (SDQ) with a sensitivity of 80.5% and a specificity of 81.3% is simple to apply using a score of 15 dysphagia-associated questions to detect PD-related dysphagia^120^. A score >10 recommends further dysphagia diagnostic (max. score 44.5). Additionally, the patient-rated Radboud Oral Motor Inventory for Parkinson’s disease (ROMP) questionnaire is used for assessment of speech, swallowing, and saliva control^121^.
2. The Munich Dysphagia Test—Parkinson’s disease (MDT-PD) with a sensitivity of 82% and a specificity of 71% was designed to detect milder forms of dysphagia without aspiration risk^122^, although its usefulness as a screening tool for aspiration events is controversial^123^. A French version is also available^124^.
3. The Non-Motor Symptoms Questionnaire (NMS-Quest; Question 3: “difficulty swallowing food or drink or problems with choking”) and the Movement Disorder Society—Unified Parkinson’s disease rating scale (MDS-UPDRS; Question 2.3 of the UPDRS II: “problems swallowing pills or eating meals”) each also include one question about swallowing difficulties^125,126^. However, in a recent study, NMS and MDS-UPDRS were identified as unreliable tools for detecting previous aspiration^127^.
4. Normal water tests that are useful for diagnosing severe dysphagia, e.g., in stroke patients, are not reliable screening tools for PD-related dysphagia when compared with instrumental diagnostic tools^115^. A detection of a ‘wet voice’ after different bolus consistencies showed a too low sensitivity to be a solid marker of penetration/aspiration in PD^128^. Therefore, a modified water test was developed to evaluate the stimulability of drinking by using a maximum performance test (maximum swallowing volume <20 mL, maximum swallowing speed <10 mL/s)^129^. Nonetheless, in a more recent study, swallowing speed was found to be prone to methodological errors and not unsuitable as a screening instrument to predict aspiration in PD patients^130^.

#### Instrumental diagnostic tools

Flexible Endoscopic Evaluation of Swallowing (FEES) and Videofluoroscopic Swallowing Study are both considered to be the gold standard for evaluating oropharyngeal dysphagia^131,132^. These instrumental tools should be applied in cases of unclear and severe PD-associated dysphagia, especially to detect silent aspiration as well as specific dysphagia phenotypes^129,133^.

#### Therapeutic management

Over the years, a number of studies have provided evidence-based recommendations for treatment of oropharyngeal dysphagia^21,101,134,135^. Swallowing therapy, especially as performed by speech and language therapists, and other specific therapeutic options might help to improve oropharyngeal swallowing impairment:

#### Pharmacotherapy

The effects of dopaminergic medication and levodopa on swallowing function and its role in dysphagia treatment are controversially discussed^136–138^. Oropharyngeal swallowing parameters with good levodopa-responsiveness could be pharyngeal residue (especially in the valleculae), penetration, and oral as well as pharyngeal transit times^139,140^. Furthermore, some studies indicate positive effects of the dopamine agonists apomorphine and transdermal rotigotine^141–143^. As such, an examination whether an improvement in swallowing function could be achieved by increasing or optimizing dopaminergic medication should be performed on a case-by-case basis, i.e., by using the FEES-Levodopa-Test. In this test, three salient parameters (premature spillage, penetration/aspiration events, and residues, each tested with liquid, semisolid, and solid food consistencies) are assessed in off- and on-stage conditions performing a specific score^140^. A score improvement of >30% indicates levodopa responsiveness of dysphagia^140^. Subsequently, in such cases, optimizing dopaminergic medication should be considered. Furthermore, levodopa-carbidopa intestinal gel (LCIG) infusion therapy might be capable of alleviating pharyngeal bradykinesia and premature bolus spillage^144^.

#### Deep brain stimulation (DBS)

To date, detailed information about the effects of DBS on swallowing function in PD patients remain limited^145,146^. Using stimulation of the subthalamicus nucleus (STN), low-frequency stimulations (i.e., 60 Hz) may have a beneficial effect on swallowing dysfunction in patients with freezing of gait^145,147^, whereas high-frequency stimulation might result in beneficial, no, or detrimental effects^145^. A short-term improvement (lower aspiration rate) was indicated as well but without confirmation in the long-term observation^148^. Simultaneous STN and substantia nigra (SNr) stimulation seem to have no additional beneficial effect on dysphagia compared with conventional STN stimulation, but swallowing function does not deteriorate as a result^149^.

#### Neuromuscular electrical stimulation (NMES)

The use of NMES in PD patients seems to have no measurable benefit^150,151^. A recent study using new electrode placement methods indicates increased hyoid bone movement and reduced aspiration risks^152^, but NMES cannot be recommended for PD dysphagia treatment at the present time.

#### Behavioral swallowing therapy

Because of heterogenous study populations and therapeutic methods as well as different outcome measures, general recommendations for non-pharmacological treatment are difficult to provide^135^. However, some therapeutic strategies are promising for individual treatment of specific patterns of PD-related dysphagia: Thickened liquids and the chin-tuck maneuver might help to prevent liquid aspiration^153,154^. The Lee Silverman Voice Treatment (LVST^®^), originally developed for treatment of PD-associated dysarthria, can also improve swallowing function, although controlled clinical trials are not yet available and the effects are unspecific^155^. With regard to therapeutic strategies, dual task situations should be avoided in real-life circumstances to focus attention on swallowing performance^112^. Two larger, randomized placebo-controlled studies showed a positive effect on swallowing safety and efficiency in PD patients who had performed a 4-week- expiratory muscle strength training regimen^156,157^. Video-assisted swallowing therapy and specific swallowing skill training using surface electromyography might also be helpful for providing biofeedback to patients^158,159^. In general, every affected PD patient should receive a detailed examination of swallowing disturbance patterns resulting in an individual training program based on available therapeutic methods. The efficacy of the method(s) selected should be confirmed via instrumental testing^133^.

#### Treatment of sialorrhea

Parkinson-related sialorrhea can be managed effectively with injections of botulinum toxin A or B into the parotid and submandibular glands^160^. Another pharmacological treatment option might be the application of the anticholinergic drug glycopyrrolate because it crosses the blood-brain barrier and therefore does not have central anticholinergic side-effects^161^. In addition, gum chewing also helps to improve PD-related sialorrhea in the short term but without maintaining a long-term effect^162^.

### Esophagogastral phase

#### Prevalence and main clinical findings

The prevalence of impaired gastric emptying in PD ranges from 70 to 100% and may be present in both early and advanced stages^6,163^. Major clinical manifestations include nausea, vomiting, early satiety, and postprandial fullness, and these can lead to weight loss, malnutrition, and dehydration^164^. Furthermore, there is growing evidence for a significant relationship between delayed gastric emptying and levodopa pharmacokinetics leading to drug-response fluctuations with delayed or missed on-phases after medication intake^165^.

Esophageal motility disorders appear to occur very early and even in premotor stages of PD^166,167^. A hypotensive peristalsis of the tubular esophagus occurs most frequently and early in the disease course, whereas in later stages diffuse esophageal spasms and multiple contractions may develop^167^. However, primary opening disorders of the upper esophageal sphincter are rare^167^.

#### Diagnostic management

Impaired gastric emptying is defined as >60% retention at 2 h postprandially and/or >10% retention at 4 h after ingestion of a radioactive technetium Tc 99m-labeled solid food^168^. Other quantitative methods are the use of breath tests with nonradioactive 13C-sodium octanoate bound into a solid meal, or real time visualization by magnetic resonance imaging and electrogastrography^164^. Because clinical evaluation is difficult, diagnostic examination of esophageal motility disorders nowadays is normally performed by using High Resolution Manometry to detect esophageal alterations^169^.

#### Therapeutic management

Therapeutic options for managing PD-associated esophageal motility disorders are rare to date. A pilot study indicates a possible usefulness of botulinum toxin injections for treatment of esophageal spasms, but more evidence is needed^170^. STN stimulation might also improve esophageal motility^171^. The use of capsaicin seems to be capable of improving esophageal motility as well as upper esophageal sphincter contraction, and it might be a promising tool for further treatment^172,173^. In gastroparesis, the increase of levodopa dosage may impair delayed gastric emptying^174^. Pharmacotherapy options using domperidone might be useful but are said to increase the risk of a long QT syndrome. Recent studies have indicated possible positive effects by using nitzatidine or ghrelin agonists but these require further evaluation^6^. Benefits from botulinum toxin injection in the pyloric sphincter and possible use of STN-DBS have been reported as well^175,176^. However, LCIG (with or without entacapone application), subcutaneous apomorphin, and the rotigotine patch are helpful solutions for bypassing the GIT and therefore could be administered in cases of clinically relevant effects of esophageal spasms as well as gastroparesis on somatomotor symptoms.

### Summary/Practical algorithm for management

Disturbances of the upper GIT in PD, especially oropharyngeal dysphagia, are complex syndromes that occur early in disease duration but often remain unnoticed until severe complications, such as aspiration pneumonia, develop. Accordingly, standardized and early diagnostic approaches as well as focused treatment of specific dysphagia patterns are required to help affected individuals. Table 2 provides a summary of the most relevant clinical manifestations of upper GIT impairment and feasible treatment approaches.

**Table 2:** Summary of most relevant clinical manifestations of upper GIT impairment and feasible treatment approaches.

Symptom	Pharmacotherapy	Swallowing therapy by Speech language therapists
Oropharyngeal freezing:	Increase dose of L-dopa before meal times Amantadine?	Triggering of swallowing reflex External triggers?
Premature spillage:		Oral bolus control Avoid dual tasks
Penetration/Aspiration:	Non-oral delivery: patch or pump?	Protective reflexes Sensory stimulation Supraglottic swallow maneuver Safe food consistencies? PEG?
Pharyngeal residues without motor fluctuations:	Individual assessment of L-dopa responsiveness, if positive: Increase dose of L-dopa before meals	Effortful swallow exercise
Pharyngeal residues without motor fluctuations:	Individual assessment of L-dopa responsiveness, if positive: Optimize oral treatment Non-oral delivery: patch or pump?	Meal times during on state condition Effortful swallow exercise in off state condition
Esophageal spasms:	Non-oral delivery: patch or pump? Botulinum toxin injections into upper esophageal sphincter?	Protective reflexes Mendelsohn swallow exercise Safe food consistencies? PEG?

## Neurology: lower GIT

### Colon

#### Prevalence of constipation

Since initially being mentioned by James Parkinson, constipation has been considered a very frequent symptom that occurs in up to 80% of PD patients^6,14,177–181^. As is often the case, constipation is described as the most frequent autonomic symptom^14,182,183^. Notably, healthy people with constipation complaints, including delayed passage of stools, hard stools, or a sensation of incomplete evacuation, have shown a greater risk for subsequently developing PD^184–186^. This fits well the neuropathological studies published by Braak and coworkers^16,17^ (see chapter 3). Constipation is currently considered one of the most relevant early signs of PD, and its frequency seems to be higher than the subjective complaints^19,184,185,187–189^. The GIT may even play an important role in PD pathogenesis^16,80,185^ and as a prognostic factor, inasmuch as a significant relationship between constipation severity and progression to dementia has recently been demonstrated^190,191^. Additional studies are needed to determine whether a similar relationship exists in patients who develop dementia with Lewy bodies (DLB).

#### Pathophysiology

Medications, reduced physical movement, a reduced muscle tone in the diaphragm and abdominal musculature, and reduced intake of fibers and liquids have been advanced as causes for constipation^14^. Beginning with the earliest studies and onwards, anticholinergic agents have been particularly related to severe constipating effects, including even the development of a megacolon. Constipation in PD is definitely disease-related^189^. It was described long before any specific therapy had been found^14,177^, and many studies of yet untreated patients were able to demonstrate delayed transit^6,14^. It is much more probable that, in PD patients, a delayed transit plays an intrinsic and prominent role, and that constipation even can be exacerbated by the medical treatment itself^14,192^.

The causes underlying the delayed transit are most probably degenerative changes involving Lewy pathology located centrally, including the spinal cord intermediolateral nucleus, and peripherally extending from the upper esophagus to the rectum in the Auerbach plexus (myenteric plexus) and Meissner plexus (submucous plexus)^3,43,47,53^. Additionally, anismus, a failure of relaxation, or involuntary contractions of the anal sphincters during defecation (extremely rare!), can lead to so-called “outlet” constipation^14^.

#### Diagnostic management

Because constipation can develop into a megacolon, pseudo-obstruction, or volvulus, adequate diagnosis is essential^14^. Unfortunately, a megacolon usually remains asymptomatic, with the exception of the singular symptom of constipation, although an ileus followed by surgery^14^ and colon perforation have been described as megalcolon consequences. Surprisingly, despite the existence of severe constipation at the time of presentation, patients seldom report this problem spontaneously, often because of embarrassment, which indicates that it is most probably underdiagnosed^189^.

**Fig. 4:**
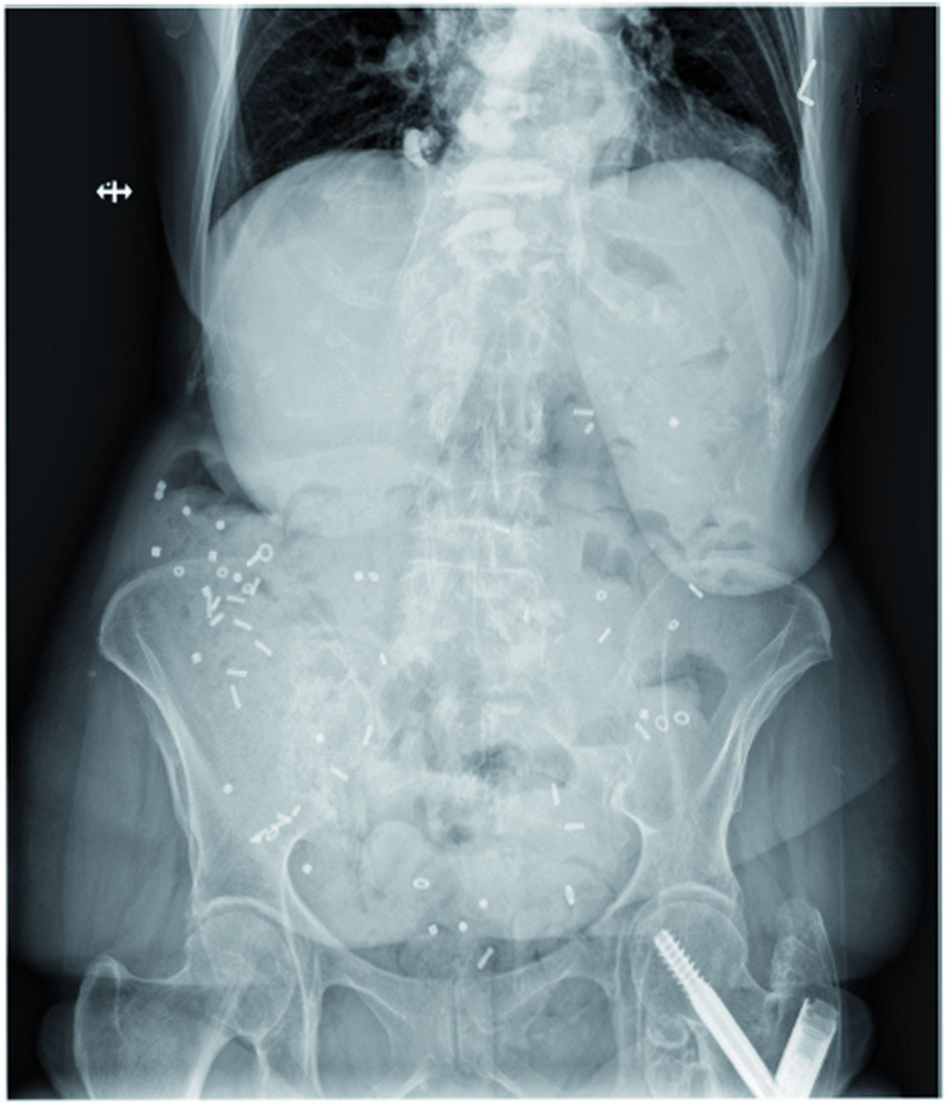
Colonic transit time in a 72-year-old male PD patient. White spots in the entire colon are radiopaque markers (erect, anterior-posterior).

A simple method during the diagnostic work-up involves administering radiopaque markers (ROM) (Fig. 4) as the gold standard diagnostic test^14^: Abdominal X-rays are taken at defined time intervals to identify the retained numbers of ingested ROMs to calculate colonic transit time. Alternatively, a gamma camera can be used to track the movements of radioisotope test meals or capsules at specified time points for quantitative evaluation of scintigraphic colonic transit times^193^. For orientation, it is also useful to ask patients to eat poppy seed cake and then note when the poppy seeds are excreted.

Although there is no gold standard method for the assessment of outlet constipation, defecography is widely preferred. It involves the instillation of barium in the rectum, and subjects are then asked to empty it during recording of a cinematic film^194^. Anorectal dysfunction can also be assessed by external anal sphincter electromyography, a balloon distension and expulsion test, and anorectal manometry^195^.

#### Therapeutic management

At present, there are no specific guidelines for the management of PD-associated constipation available. A fiber-rich diet, psyllium as a bulk laxative, stool softener, and sufficient liquid-intake have high therapeutic value in treating constipation, but regular physical exercise and physical therapy are also advisable^178^. Exclusion of aggravating factors, such as anticholinergics, should be considered^178^. Unfortunately, these measures are only useful in mild or moderate cases. In many instances, a colonic transit of more than 7 days is reported, and no improvement of colonic transit can be achieved by the various therapeutic options owing to the upper threshold. In this case, additional medication must be prescibed. An effect of domperidone in the upper GIT has not been shown for constipation^178^.

Stimulants, such as bisacodyl, sodium picosulfate, and senna are safe and helpful^196^. In addition, stimulant laxatives and osmotic laxatives are recommended. The best results to date are achieved with macrogol^197,198^. A disadvantage of lactulose is flatulence^14^. Positive data are also available for therapies with probiotics and probiotic fibers^196,199^.

There are still no studies on the effects of modern prokinetic agents, such as serotonin (5-HT4) agonists, e.g., mosapride^200^. In the meantime, prucaloprid^201^ has been approved for severe constipation and may be administered to PD patients, although specific studies in this population are still lacking. Several new drugs, including relamorelin (ghrelin agonist)^181^, and chlorid channel activators, such as linaclotide, lubipprostone, and plenacanatide^188,201–203^ are in discussion. In rare cases of anismus, we recommend botulinum toxin injections^14^.

## Excursus: the role of the GIT microbiome

The GIT microbiome in PD has been intensively researched in recent years^6,30,37,38,204–210^. By applying metagenomic and next-generation sequencing procedures, it is now possible to distinguish PD patients from healthy individuals^204,210^ at a very early disease stage by means of individually altered microbiota^204^. There may even be a ‘prodromal’ GIT microbiome because a microbial shift has been found, for instance, in patients with RBD^211^. In one large cohort, reduced GIT microbial diversity in PD patients correlated significantly with greater GIT symptom severity in comparison to controls^212^, and evidence exists for an ‘enteric pro-inflammatory profile’ in PD^213,214^. Intestinal dysbiosis and small intestinal bacterial overgrowth in PD patients^215^ might increase intestinal barrier permeability, thereby triggering excessive stimulation of the innate immune system and systemic inflammation, mechanisms possibly involved in the initiation of α-synuclein deposition^216–218^. According to this scenario, α-synuclein expression in the GIT would reflect an immune defense mechanism^219^, which is further supported by the finding that the protein is capable of triggering T cell responses that may also potentiate neurodegeneration^220^.

At present, interpretation of the available findings is difficult because a great variety of factors can influence the microbial configuration of the GIT. For example, evaluation of the GIT microbiome in patients undergoing treatment for PD is still of limited use, inasmuch as levodopa and other antiparkinson medications act upon the intestinal flora^192,206,221^, and, at least in a subset of patients, the opposite is also the true^222^. In addition, particularly for PD, it cannot be clarified retrospectively whether the altered GIT microbiome is the cause or the effect of motility disturbances, such as severe constipation^30,207,223^, and the association between the microbiome and neuroinflammation in PD still remains unclear^63,204,206,224^, in part because the cohorts studied to date, with few notable exceptions^222,225^, have been small^204,207^. Finally, if the microbiome and its metabolites were to play a key pathogenetic role in PD, then considerable differences should be observable between populations on different continents owing simply to dietary variability, but this has not proved to be the case. Nonetheless, it is imperative, going forward, to examine not only the precise role of the GIT microbiome and the effects a targeted diet and probiotics might have on PD patients^196,226,227^ but also the potential advantages and adverse side effects associated with fecal microbiotica transplantation^228–231^.

## Final conclusions/practical algorithm for management

Dysfunction of the upper GIT in PD, especially oropharyngeal dysphagia, are complex syndromes occurring early in disease that often remain unnoticed until severe complications, such as aspiration pneumonia, become manifest. In the lower GIT, constipation is a widespread and debilitating symptom with the potential of leading to severe bowel complications and even cognitive dysfunction.

In closing, standardized and early diagnostic approaches together with continuous and long-term treatment are necessary to help patients (Table 2).

### Reporting summary

Further information on research design is available in the Nature Research Reporting Summary linked to this article.

## Supplementary information

Reporting Summary Checklist

## Data Availability

All data generated or analyzed during this study are included in this published review (see references).
